# Picking up the bill - improving health-care utilisation in the Democratic Republic of Congo through user fee subsidisation: a before and after study

**DOI:** 10.1186/s12913-014-0504-6

**Published:** 2014-11-05

**Authors:** Rishma Maini, Rafael Van den Bergh, Johan van Griensven, Katie Tayler-Smith, Janet Ousley, Daniel Carter, Seb Mhatre, Lara Ho, Rony Zachariah

**Affiliations:** Department for International Development, British Embassy, 83 Ave Roi Baudouin, Kinshasa, the Democratic Republic of Congo; Médecins Sans Frontières Operational Centre Brussels, Operational Research Unit (LuxOR), Luxembourg, Luxembourg; Institute of Tropical Medicine, Antwerp, Belgium; International Rescue Committee, Kinshasa, the Democratic Republic of Congo; Department for International Development, London, UK

**Keywords:** User fees, Subsidisation, Health-care utilisation, Operational research, DRC

## Abstract

**Background:**

User fees have been shown to constitute a major barrier to the utilisation of health-care, particularly in low-income countries such as the Democratic Republic of Congo (DRC). Importantly, such barriers can lead to the exclusion of vulnerable individuals from health-care. In 2008, a donor-funded primary health-care programme began implementing user fee subsidisation in 20 health zones of the DRC. In this study, we quantified the short and long-term effects of this policy on health-care utilisation.

**Methods:**

Sixteen health zones were included for analysis. Using routinely collected health-care utilisation data before and after policy implementation, interrupted time series regression was applied to quantify the temporal impact of the user fee policy in the studied health zones. Payment of salary supplements to health-care workers and provision of free drugs - the other components of the programme - were controlled for where possible.

**Results:**

Fourteen (88%) health zones showed an immediate positive effect in health-care utilisation rates (overall median increase of 19%, interquartile range 11 to 43) one month after the policy was introduced, and the effect was significant in seven zones (*P* <0.05). This initial effect was sustained or increased at 24 months in five health zones but was only significant in one health zone at *P* <0.05. Utilisation reduced over time in the remaining health zones (overall median increase of 4%, interquartile range −10 to 33). The modelled mean health-care utilisation rate initially increased significantly from 43 consultations/1000 population to 51 consultations/1000 population during the first month following implementation (*P* <0.01). However, the on-going effect was not significant (*P* =0.69).

**Conclusions:**

Our research brings mixed findings on the effectiveness of user fee subsidisation as a strategy to increase the utilisation of services. Future work should focus on feasibility issues associated with the removal or reduction of user fees and how to sustain its effects on utilisation in the longer term.

## Background

User fees, defined as out-of-pocket payments by patients for medical services in health facilities, were introduced in many African countries as a response to deteriorating primary health-care systems and a decline in both donor and government expenditure on health during the global economic crisis of the 1980s [1]. This was supported both by the United Nations Children's Fund (UNICEF) and the World Health Organization through the Bamako Initiative [2], which aimed to secure the continuation of the delivery of basic services through generating funds from communities by charging drugs at a mark-up cost. The additional funds generated through this mark-up could then be used by the community to establish a revolving drug fund and finance other primary health-care services in combination with government and donor funding. The initiative also sought to improve accountability between providers and communities, as well as provide a sustainable way of improving access to health facilities, health-care utilisation and quality of care, while lowering frivolous use of health services [3,1].

However, the implementation of debt relief initiatives for developing countries [4], reinvestment of donors in the health sector, strong economic growth, and the Millennium Development Goals have created a new context. Once seen as a solution, user fees are growingly seen as part of the problem. Ponsar *et al*. [5] showed that user fees in several African countries may present significant barriers for accessing health-care and can result in the exclusion of vulnerable individuals. They suggested a mechanism of user fee abolition combined with compensation of health facilities for the lost revenue. User fee subsidisation – whereby the running cost of services (which covers fuel for generators, disinfectant products, and medical supplies) is subsidised by a donor and/or government thus resulting in a lower fee to the user - may be an expedient approach towards achieving such abolition, particularly for vulnerable groups.

Yet there is still an ongoing debate regarding user fees in the literature. Evidence has shown that introducing or increasing user fees can result in a decrease in the use of both preventative and curative health-care services [6], while abolishing user fees can lead to an overall increase in health-care utilisation rates [7]. However, it should also be recognised that failure to support the supply-side of health-care may constrain the value of user fee removal [8,9]. In areas where user fees have been reduced or abolished, the resultant increase in health-care utilisation can negatively affect staff morale because of the increased workload and reduced revenue [10,11]. In addition, although the evidence is limited, the abolishment of user fees has sometimes been accompanied by an increase in the number of reports of informal payments being made to health workers [12]. These findings illustrate the importance of monitoring the implementation of policies targeting user fees.

To date, qualitative studies on patients’ perceptions of user fees have been previously conducted in the Democratic Republic of Congo (DRC) [5] but there have been no quantitative studies on user fee subsidisation in this context. This study aimed to address this gap by answering the following research question: what are the effects on health-care utilisation of a policy to subsidise user fees in the DRC? Specific objectives included assessing the short-and long-term effects of user fee subsidisation on health-care utilisation in a) individual health zones and b) for the entire sample population. It is hoped that the findings of this study will contribute to discussions on whether user fees should be subsidised through third party subsidisation in the DRC and other low-income countries [13].

## Background

### The DRC

The DRC has faced decades of conflict and instability which continues to impact on its health system. Health services rely on a system of cost recovery through user fees, as government financing of the health sector is very limited [14]. Yet, according to the 2011 Human Development Index, the DRC is the poorest country in the world [15]. It has the lowest Gross Domestic Product per capita in the world with 60% of its population living on less than $1.25 per day [16]. In such a context, user fees would likely constitute a major barrier for accessing health services.

The country also has catastrophic health indicators, and child and maternal mortality rates are respectively the second and fourth highest in the world [17,18]. Utilisation of health services is extremely low; on average one person consults health services every 6.7 years [19].

The DRC government published its Health Systems Strengthening Strategy in 2006 [20], which recognised the poor budgetary allocation to health and the weak execution of these funds. The strategy proposed different options for improving the health financing system such as increasing the mobilisation of public sector resources, use of community health insurance schemes and also user fee subsidisation. The DRC Ministry of Health even has a policy to reduce or abolish fees for some “vulnerable groups” such as sexual violence survivors, indigents and the elderly, and supports the abolishment of user fees during emergency periods in conflict zones, but this is variably enforced.

### Access to health-care programme

Between 2008 and 2012, two non-governmental organisations (NGOs) - the International Rescue Committee (IRC) and Medical Emergency Relief International (MERLIN) - implemented the Access to Health-Care Programme in the DRC which was funded by the Department of International Development (DFID) [21]. The programme covered a total of 20 health zones and was located in four provinces – Province Orientale, Maniema, Kasai Occidental, and South Kivu, the latter of which has been affected by devastating conflict since the second Congo war, which occurred between 1998 and 2003 [22]. Each health zone is similar to a “health district” in other areas of Africa; it is a well-defined geographical area comprising a referral hospital and satellite health centres serving a population of approximately 120,000 people.

The Access to Health-Care Programme supported public primary health-care centres and hospitals situated in these health zones to provide a package of primary health-care services, covering a total population of approximately two and a half million inhabitants. The support included financing of: day-to-day running costs, construction and rehabilitation of facilities, medical equipment, training of health workers, drugs, and salary supplements for health workers. The cost per capita per year (which included NGO costs) was estimated to be $13.9.

Fixed amounts of money were paid by the NGOs to health facilities each month for their running costs. For health centres, the average amount was $40 per month, while $1500 was given to hospitals, yielding a cost per capita per year of $0.20. Given the general disrepair of many facilities, health facilities were constructed or rehabilitated where necessary. In order to improve the capacity of health personnel, the NGOs provided clinical, management, monitoring, evaluation and community health training throughout the programme. Including equipment and NGO costs, this resulted in a high cost per capita per year of approximately $10.15.

In terms of drug provision, the NGOs operated a “pull” system; health zones placed orders with the NGOs which were based on average monthly drug consumption calculations. The amount spent by NGOs on drugs was estimated to be $1.5 per capita per year, which included transportation costs. Staff were discouraged from over-reporting drug consumption as regular and random supervisory drug checks were conducted by the NGOs on a monthly basis. Another deterrent to over-reporting was that several registers would need to be changed in order to record the distribution of drugs; these included the patient register, dispensing register and drug report. Frequent audits of the drug management systems were undertaken in order to verify that these registers were consistent with one another.

With respect to salary supplements, seventy percent of the salary supplement was provided to workers each month based on their attendance at health facilities, while the remaining thirty percent was dependent on attaining a minimum score at the quarterly performance review of the health facility. The amount allocated to each health zone per month also varied according to the number of health facilities and number and grades of health workers; the average cost per capita per year was approximately $2.05.

Drug provision, salary supplements, and the financing of running costs in order to subsidise user fees were introduced progressively at different times in health zones, with all interventions in place in all health zones by October 2010 (see Table 1). In general, free provision of drugs either preceded the introduction of salary supplements or occurred simultaneously, while the financing of running costs occurred some months thereafter. Only once the running costs were financed were the user fees then lowered. Financing of all of these costs by DFID enabled all primary health-care services to be provided free of charge (i.e. fully subsidised) for vulnerable groups which included: pregnant women, children under five years of age, survivors of sexual violence, and indigents. It also allowed services to be provided at a substantially reduced cost (i.e. partially subsidised) of 30 cents for the rest of the population. Prior to the programme, the cost for a consultation was just over $5, equivalent to the average weekly wage, with additional charges for drugs, tests and procedures. No other donor-funded health systems strengthening programmes were known to be operating within the health zones specified above during the Access to health-care programme.

**Table 1:** **Timing of interventions in each of the health zones**

**Province**	**Health zone**	**Drug supply for free by the project to the health centres**	**Salary supplement paid to the staff**	**Subsidisation of running costs and userfee subsidisation (full and partial)**	**First month of the data set**	**Last month of the dataset**	**Regression equation**
Kasai Occidental	Demba	01/04/2008	01/04/2008	01/04/2009	January 2009	December 2012	1
South Kivu	Itombwe	01/04/2008	01/04/2008	01/09/2010	January 2009	December 2012	1
Maniema	Kailo	01/04/2008	01/08/2008	01/08/2010	January 2009	December 2012	1
South Kivu	Kabare	01/04/2008	01/04/2008	01/09/2010	January 2009	December 2012	1
Maniema	Kampene	01/04/2008	01/08/2008	01/08/2010	January 2009	December 2012	1
South Kivu	Minembwe	01/04/2008	01/04/2008	01/09/2010	January 2009	December 2012	1
Maniema	Pangi	01/04/2008	01/08/2008	01/08/2010	January 2009	December 2012	1
South Kivu	Kalehe	01/04/2008	01/04/2008	01/08/2010	January 2009	December 2012	1
Province Orientale	Ubundu	01/04/2008	01/04/2008	01/04/2009	March 2008	December 2012	1
Maniema	Punia	01/04/2008	01/08/2008	01/08/2010	January 2008	December 2012	2
Maniema	Ferekeni	01/04/2008	01/08/2008	01/08/2010	January 2008	December 2012	2
Maniema	Kalima	01/04/2008	01/08/2008	01/08/2010	January 2008	December 2012	2
Maniema	Lubutu	01/04/2008	01/08/2008	01/08/2010	January 2008	December 2012	2
Maniema	Obokote	01/04/2008	01/08/2008	01/08/2010	January 2008	December 2012	2
Province Orientale	Banalia	01/10/2008	01/10/2008	01/04/2009	March 2008	December 2012	3
Province Orientale	Bengamisa	01/10/2008	01/10/2008	01/04/2009	March 2008	December 2012	3

N.B For health zones Alunguli, Kindu, Lukonga and Mutoto, data included consultations at private facilities or the times at which salary supplements or free drug provision were introduced were not known. As a result, these zones were not analysed in the study. The remaining 16 health zones had less than 10% of data missing. All health zones in the table above had 12 months data prior to user fee subsidisation. The last seven zones had at least three or more data points prior to the introduction of drugs and salary supplements.

## Method

### Design

This was a “before and after” study assessing the effects of user fee subsidisation on health-care utilisation.

### Data collection

All patients attending public primary health-care facilities supported by the Access to Health-Care Programme were included in this study. Data on the number of primary health-care consultations were extracted from the national routine health information system, known as the *Système National d’Informations Sanitaires*, for each month between January 2008 and December 2012, by health zone. For all zones, data on health-care utilisation were cross-checked against health facility registers on a bi-annual basis between 2008 and 2012, in order to confirm that reporting was accurate. Spot checks in all health zones were also conducted on a quarterly basis for a small number of indicators selected at random; these indicators were either the overall utilisation rate, the number of malaria consultations, the number of assisted births, or the number of vaccinations. Similar to the bi-annual checks, data on the routine health information system were cross-checked against registers held at the facility.

All health zones had at least 12 months of data prior to the introduction of user fee subsidies. Seven out of 16 health zones had at least three or more data points prior to the introduction of drugs and salary supplements.

### Data analysis

Statistical analyses were performed using the software “R”, version 3.0. The main outcome measure was the health-care utilisation rate per 1000 population (the number of monthly consultations divided by the annual population for each health zone multiplied by 1000). Population denominators within health zones were extrapolated from the 1984 census (the most recent available) [23], with population growth assumed to be 2.7% per annum [24]. Although this source of data is thirty years out of date, it continues to be used by the Ministry of Health in all health information data calculations. Subsequent census data for health zones have been collected during vaccination campaigns but have never been validated and so were not used.

For each of the health zones, interrupted time series regression analysis was used to adjust for structural trends and potential serial correlation of the data, as described in detail by Lagarde [25]. In order to aggregate the data and understand the overall effect of subsidies on utilisation, all monthly data points for each health zone 12 months prior to the introduction of user fee subsidies and 24 months following the introduction of user fee subsidies were compiled and the mean utilisation rate at each of these time points calculated.

Two econometric models were used; one included dummy variables for salary supplements and drugs, and the other did not. For the nine health zones where there were no data points prior to the introduction of drugs and salary supplements (see Table 1), equation 1 was used:

1 $$ {\mathbf{Y}}_{\mathbf{t}}\kern0.5em =\kern0.5em {\boldsymbol{\upbeta}}_{\mathbf{o}}\kern0.5em +\kern0.5em {\boldsymbol{\upbeta}}_{\mathbf{1}}\kern0.5em *\kern0.5em \mathbf{time}\kern0.5em +\kern0.5em {\boldsymbol{\upbeta}}_{\mathbf{2}}\kern0.5em *\kern0.5em \mathbf{subsidies}\kern0.5em +\kern0.5em {\boldsymbol{\upbeta}}_{\mathbf{3}}\kern0.5em *\kern0.5em \mathbf{postslope}\kern0.5em +\kern0.5em {\boldsymbol{\upvarepsilon}}_{\mathbf{t}} $$

In this equation, Y_t_ represents health-care utilisation at time t, where t is a continuous variable indicating time in months which is coded sequentially from 0 from the start of the programme until the end of the programme. User fee subsidies are coded 0 prior to the intervention and then 1 for all post-intervention time points. The postslope is also coded 0 for time points prior to the intervention, and then coded sequentially from 1 when the intervention is introduced. β_o_ represents the constant which captures the baseline level of the outcome at time 0, while β_1_ estimates the structural trend of the data and is independent of the intervention. β_2_ estimates the immediate impact of the intervention and β_3_ estimates the change in trend after the intervention is introduced. This equation was also used to measure the aggregate effect of subsidies, using the mean utilisation rate across all health zones.

Essentially, the difference before and after the introduction of user fee subsidisation was quantified by testing the change in the level (β_2_) and the slope (β_3_) of the regression. A change in level between the pre- and post-intervention segments indicated an immediate (short-term) effect, and a change in slope indicated a change in trend and therefore on-going (long-term) effect.

Drugs and salary supplements were included as dummy variables in the interrupted time series analysis model for the seven zones in which data points prior to their introduction were available (see Table 1). The aim was to adjust for the effects of these potential confounding factors in those zones. Where drugs and salary supplements were introduced at the same time, equation 2 was used:

2 $$ \begin{array}{l}{\mathbf{Y}}_{\mathbf{t}}\kern0.5em =\kern0.5em {\boldsymbol{\upbeta}}_{\mathbf{o}}\kern0.5em +\kern0.5em {\boldsymbol{\upbeta}}_{\mathbf{1}}\kern0.5em *\kern0.5em \mathbf{time}\kern0.5em +\kern0.5em {\boldsymbol{\upbeta}}_{\mathbf{2}}\kern0.5em *\kern0.5em \mathbf{salary}\ \mathbf{supplements}\ \mathbf{and}\ \mathbf{drugs}\kern0.5em +\kern0.5em {\boldsymbol{\upbeta}}_{\mathbf{3}}\kern0.5em *\kern0.5em \mathbf{subsidies}\kern0.5em +\kern0.5em {\boldsymbol{\upbeta}}_{\mathbf{4}}\kern0.5em *\kern0.5em \mathbf{postslope}\ \mathbf{salary}\\ {}\mathbf{supplements}\ \mathbf{and}\ \mathbf{drugs}\kern0.5em +\kern0.5em {\boldsymbol{\upbeta}}_{\mathbf{5}}\kern0.5em *\kern0.5em \mathbf{postslope}\ \mathbf{subsidies}\kern0.5em +\kern0.5em {\boldsymbol{\upvarepsilon}}_{\mathbf{t}}\end{array} $$

In this equation, similar to user fee subsidies, salary supplements and drugs are coded 0 prior to their introduction and then 1 for all post-intervention time points. Postslope salary supplements and drugs is also coded 0 for time points prior to their implementation, and then coded sequentially from 1 when they have been introduced.

Where drugs and salary supplements were introduced at different times, equation 3 was employed:

3 $$ \begin{array}{l}{\mathbf{Y}}_{\mathbf{t}}\kern0.5em =\kern0.5em {\boldsymbol{\upbeta}}_{\mathbf{o}}\kern0.5em +\kern0.5em {\boldsymbol{\upbeta}}_{\mathbf{1}}\kern0.5em *\kern0.5em \mathbf{time}\kern0.5em +\kern0.5em {\boldsymbol{\upbeta}}_{\mathbf{2}}\kern0.5em *\kern0.5em \mathbf{drugs}\kern0.5em +\kern0.5em {\boldsymbol{\upbeta}}_{\mathbf{3}}\kern0.5em *\kern0.5em \mathbf{salary}\ \mathbf{supplements}\kern0.5em +\kern0.5em {\boldsymbol{\upbeta}}_{\mathbf{4}}\kern0.5em *\kern0.5em \mathbf{subsidies}\kern0.5em +\kern0.5em {\boldsymbol{\upbeta}}_{\mathbf{5}}\kern0.5em *\kern0.5em \mathbf{postslope}\\ {}\mathbf{salary}\ \mathbf{supplements}\kern0.5em +\kern0.5em {\boldsymbol{\upbeta}}_{\mathbf{6}}\kern0.5em *\kern0.5em \mathbf{postslope}\ \mathbf{drugs}\kern0.5em +\kern0.5em {\boldsymbol{\upbeta}}_{\mathbf{7}}\kern0.5em *\kern0.5em \mathbf{postslope}\ \mathbf{subsidies}\kern0.5em +\kern0.5em {\boldsymbol{\upvarepsilon}}_{\mathbf{t}}\end{array} $$

As in equation two, salary supplements and drugs are coded 0 prior to their introduction and then 1 following their implementation. Postslope drugs and postslope salary supplements are also coded 0 for time points prior to their introduction, and then coded sequentially from 1 following their introduction.

For each regression model, autocorrelation of the data was detected using the Durbin-Watson test and corrected using general least squares regression [25,6].

A Student’s t-test was used to assess whether the provision of drugs or salary supplements significantly altered the effect of user fee subsidisation on health-care utilisation rates. Specifically, the test examined for differences in the mean user fee subsidisation coefficients (both the level and the slope coefficients) between zones according to their drugs and salary supplements status.

The coefficients for user fee subsidisation (level and slope) obtained from the interrupted time series regression analyses were also used to model outcomes at one and 24 months after the introduction of subsidisation, and compared to counterfactual outcomes (i.e. the projected outcomes in the absence of an intervention) as described previously by Lagarde *et al*. [7]. For example, if by month 24 of data collection the intervention has been in place for 12 months, the expected utilisation rate can be calculated by imputing the values of the explanatory variables into the estimated regression equation as follows:

$$ {\widehat{\mathbf{Y}}}_{\mathbf{2}\mathbf{4}\left(\mathbf{m}\right)}\kern0.5em =\kern0.5em {\boldsymbol{\upbeta}}_{\mathbf{o}}\kern0.5em +\kern0.5em {\boldsymbol{\upbeta}}_{\mathbf{1}}\kern0.5em *\kern0.5em \mathbf{24}\kern0.5em +\kern0.5em {\boldsymbol{\upbeta}}_{\mathbf{2}}\kern0.5em *\kern0.5em \mathbf{1}\kern0.5em +\kern0.5em {\boldsymbol{\upbeta}}_{\mathbf{3}}\kern0.5em *\kern0.5em \mathbf{1}\mathbf{3} $$

The counterfactual outcome is obtained using the following equation:

$$ {\widehat{\mathbf{Y}}}_{\mathbf{24}\left(\mathbf{c}\right)}\kern0.5em =\kern0.5em {\boldsymbol{\upbeta}}_{\mathbf{o}}\kern0.5em +\kern0.5em {\boldsymbol{\upbeta}}_{\mathbf{1}}\kern0.5em *\kern0.5em \mathbf{24} $$

The difference between these outcomes can then be calculated to obtain a relative percentage change:

$$ \Phi \kern0.5em =\kern0.5em \left({\widehat{\mathbf{Y}}}_{\mathbf{24}\left(\mathbf{m}\right)}\kern0.5em \mathbf{\hbox{-}}\kern0.5em {\widehat{\mathbf{Y}}}_{\mathbf{24}\left(\mathbf{c}\right)}\right)/{\widehat{\mathbf{Y}}}_{\mathbf{24}\left(\mathbf{c}\right)} $$

### Ethical approval

This study met the *Médecins Sans Frontières* (Geneva, Switzerland) Ethics Review Board-approved criteria for analysis of routinely-collected program data, and was also approved by the Ethics Advisory Group of the International Union Against Tuberculosis and Lung Disease (Paris, France). Ethics approval was also obtained from the Kinshasa School of Public Health (Kinshasa, DRC).

## Results

First, we will present some descriptions on the trend of utilisation rates in individual health zones. Then we will detail the significance of the coefficients for the short- and long-term effects of subsidies on utilisation at the health zone level, and at the aggregate level. Eventually, we report the relative percentage changes in utilisation at 1 month and 24 months at the health zone level.

### Trends of utilisation rates: individual and aggregate level

In all health zones, utilisation rates were higher by the end of the programme compared to the start of the programme. The health zone Banalia demonstrated the most change during the programme, with utilisation rates starting at 5 consultations/month/1000 population and ending at 63 consultations/month/1000 population. However, Kabare health zone demonstrated the least change overall during the course of the programme, with utilisation rates starting from 60 consultations/month/1000 population in 2008 and ending with only 65 consultations/month/1000 population by the end of the programme. With respect to the aggregate data, the mean utilisation rate increased from 38 consultations/month/1000 population in 2008 to 69 consultations/month/1000 population after 12 months of the policy.

### Short- and long-term effects of user fee subsidisation on health-care utilisation: Individual health zones and at aggregate level

**Figure 1:**
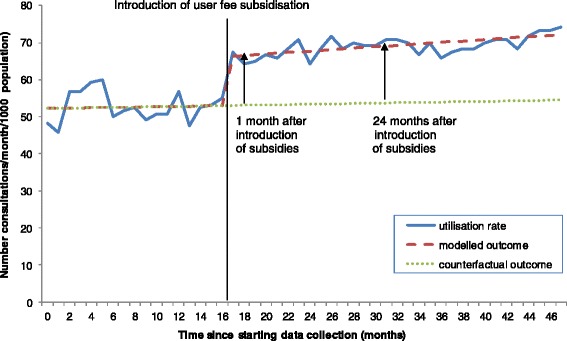
**Trend of health-care utilisation rates before and after the introduction of user fee subsidisation for one health zone in the DRC (2008 to 2012).**

Figure 1 is an example of the results observed in one health zone, and also illustrates the difference between the change in utilisation after one month and 24 months of user fee subsidisation. Similar analyses were performed for the remaining 15 health zones (see Table 1 for information on the equations used for each zone and Tables 2 and 3 for regression outputs). Seven out of the 16 health zones showed significant positive coefficients for change in level subsidies β_2_ at *P* <0.05 (short-term effect), while only Obokote health zone showed a significant positive coefficient for the change in slope subsidies (long-term effect) β_3_ at *P* <0.05.

**Table 2:** **Regression outputs for zones using equations** **1** **and** **2**

**Health zone**	**Constant (β**_**0**_ **)**	**Secular trend (β**_**1**_ **)**	**Change in level salary supplements (β**_**2**_ **)**	**Change in slope salary supplements (β**_**3**_ **)**	**Change in level drugs (β**_**2**_ **)**	**Change in slope drugs (β**_**3**_ **)**	**Change in level subsidies (β**_**2**_ **)**	**Change in slope subsidies (β**_**3**_ **)**	**Number of observations**	**Adjusted R** ^**2**^	**Durbin satson statistic**
Demba	52***	0.054	N/A	N/A	N/A	N/A	13***	0.14	48	0.88	1.5*
	(4.3)	(−0.2)					(−2.3)	(−0.22)			
Itombwe	26***	0.32	N/A	N/A	N/A	N/A	16***	0.077	48	0.86	1.68
	(−5.8)	(−0.25)					(−3.5)	(−0.3)			
Kailo	29**	0.79	N/A	N/A	N/A	N/A	8.9	−0.36	48	0.76	1.35**
	(−9.2)	(−0.4)					(−5.2)	(−0.48)			
Kabare	18	0.97	N/A	N/A	N/A	N/A	7.4	−1	48	0.67	0.82***
	(−14)	(−0.56)					(−5.9)	(−0.78)			
Kampene	22	0.89	N/A	N/A	N/A	N/A	−2.9	−0.84	48	0.06	1.21***
	(−16)	(−0.7)					(−8.8)	(−0.86)			
Minembwe	27**	0.49	N/A	N/A	N/A	N/A	7.4	0.22	48	0.78	1.56*
	(−7.9)	(−0.34)					(−4.9)	(−0.41)			
Pangi	26***	0.46*	N/A	N/A	N/A	N/A	23***	−0.38	48	0.87	2.14
	(−5)	(−0.22)					(−3.1)	(−0.25)			
Kalehe	45*	3	N/A	N/A	N/A	N/A	6.2	−0.31	48	0.32	1.05***
	(−17)	(−0.72)					(−8)	(−0.94)			
Ubundu	32	0.227	N/A	N/A	N/A	N/A	5.09	0.586	57	0.82	0.73***
	(−896)	(−1.48)					(−4.96)	(−1.65)			
Punia	21	2.4	−3.5	−1.6	1.3	0.45	18*	0.68	60	0.82	0.9***
	(−16)	(−5.7)	(−8.7)	(0.45)	(−10)	(−0.7)	(−8.2)	(0.9)			
Ferekeni	25	5.7	−2.2	0.27	−12	−5.3	9.8	−0.31	60	0.78	1.23***
	(−11)	(−4.6)	(−6.9)	(−0.32)	(−9)	(−6)	(−5.7)	(−0.46)			
Kalima	53	−1.9	1.1	10	17**	−7.5	11**	−0.31	60	0.93	1.33***
	(−6.9)	(−2.9)	(−4.4)	(−2.1)	(−5.8)	(−3.9)	(−3.4)	(−0.27)			
Lubutu	49	−1.6	−11	−6.3	−4.5	7.3	18**	0.66	60	0.44	1.39***
	(−12)	(−5.3)	(−8)	(−3.7)	(−11)	(−7)	(−6.3)	(−0.48)			
Obokote	38	−0.69	−2.2	3.1	7.9	−2	21.9***	1**	60	0.56	1.92
	(−13)	(−5.9)	(−8.2)	(−3.9)	(−13)	(−7)	(−5.2)	(−0.36)			

*P < 0.05. **P < 0.01. ***P < 0.001.

N.B. All Standard Errors are in parenthesis.

**Table 3:** **Regression output for health zones using equation** **3**

**Health zone**	**Constant (β**_**0**_ **)**	**Secular trend (β**_**1**_ **)**	**Change in level salary supplements and drugs (β**_**2**_ **)**	**Change in slope salary supplements and drugs (β**_**3**_ **)**	**Change in level subsidies (β**_**2**_ **)**	**Change in slope subsidies (β**_**3**_ **)**	**Number of observations**	**Adjusted R** ^**2**^	**Durbin watson statistic**
Bengamisa	−1.3	2	−1.1	2	9.8	−3.5	58	0.87	0.82***
	(−14)	(−2)	(−7.5)	(−3.3)	(−6.5)	(−2.2)			
Banalia	11	1.4	1.9	1.5	7	−2.4	58	0.89	1.15***
	(−12)	(−1.8)	(−6.9)	(−3)	(−5.9)	(−2)			

***P < 0.001.

N.B. All Standard Errors are in parenthesis.

Only in Kalima health zone did the introduction of drugs appear to have a significant short-term effect on utilisation (change in levels drugs β_2_ at *P* <0.01). However, no statistical difference at the *P* <0.05 level was detected between mean user fee subsidisation coefficients (mean change in level subsidies β_2_ and mean change in slope subsidies β_3_) obtained from zones which included salary supplements and drugs in the regression model, compared to those which did not. This seems to indicate that the timing of the provision of drugs and salary supplements did not significantly alter the effect of user fee subsidisation on health-care utilisation.

**Figure 2:**
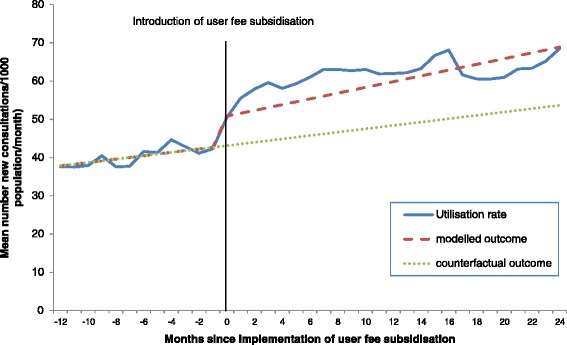
**Effects of user fee subsidisation on mean health-care utilisation rate 12 months prior to and 24 months following their introduction for 16 health zones of the DRC (2008 to 2012).**

For the effect at the aggregate level, the mean health-care utilisation rate of the 16 health zones was calculated 12 months prior to and 24 months following the introduction of user fee subsidisation (see Figure 2 below and the regression output in Table 4). There was an immediate increase in the mean health-care utilisation rate once subsidisation was introduced and the trend over time was also positive. The interrupted time series model showed that the short-term effect (change in level subsidies β_2_) was significant, with utilisation increasing from 43 consultations/1000 population to 51 consultations/1000 population (*P* <0.01). Although the relative increase in utilisation rose by 28% at 24 months, the change in slope subsidies β_3_ (long-term effect) was not significant even at the *P* <0.2 level.

**Table 4:** **Regression output for mean utilisation rate using equation** **1**

**All 16 zones**	**Constant (β**_**0**_ **)**	**Secular trend (β**_**1**_ **)**	**Change in level (β**_**2**_ **)**	**Change in slope (β**_**3**_ **)**	**Number of observations**	**Adjusted R** ^**2**^	**Durbin watson statistic**
Overall mean utilisation rate	37**	0.436	7.43**	0.312	37	0.95	0.96***
	(13.2)	(0.63)	(2.22)	(0.787)			

**P < 0.01. ***P < 0.001.

N.B. All Standard Errors are in parenthesis.

### Relative percentage changes in utilisation rates for health zones

**Figure 3:**
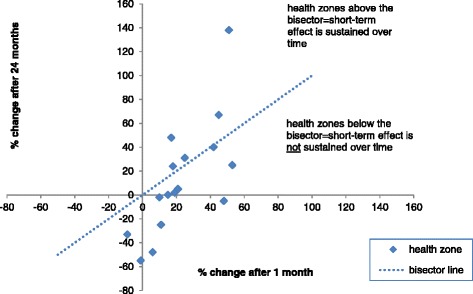
**Percentage change in health-care utilisation rates between modelled and counterfactual outcomes, one month and 24 months after introducing user fee subsidisation in the DRC (2008 to 2012).**

Figure 3 shows the relative percentage changes in utilisation rates between modelled outcomes and counterfactual outcomes at one and 24 months for each health zone (although the majority of changes were not significant as indicated by Tables 2, 3 and 4). At one month, relative changes in health-care utilisation rates ranged from −9 to 53% (median change 19%, interquartile range 11- 43%), and a positive effect was observed in all but two (12.5%) of the zones. Amongst zones with a positive effect at one month, the relative change in health-care utilisation rates at 24 months ranged from −55 – 138% (median change 4%, interquartile range 10 - 33%). Initial positive effects at one month were sustained or increased in five health zones at 24 months (zones above the bisector line) but diminished over time in the remainder.

## Discussion

This study is one of the few to quantify both the short- and long-term effects of user fee subsidisation on health-care utilisation on a large-scale, and in a fragile and conflict-affected state [6,7,26,27]. Improved coverage and therefore uptake of health-care is important as it is has been shown to be linked to improvements in health outcomes [28].

Our research brings mixed findings on the effectiveness of the user fee subsidisation as a strategy to increase the utilisation of services. On one hand, it shows that subsidising or removing user fees can result in an increase in health-care utilisation in the short-term, a finding which is consistent with other studies [6,7,29,30]. However, on the other hand, it also seems that the user fee subsidisation did not generate the long-term positive effect one could expect: in only one health zone, there was a significant positive change in the trend of utilisation. In other health zones, the change was most of the time negative but always not significant. These findings suggested that the studied user fee subsidisation sometimes generated some quick wins (significant in 7/16 health zones), but without triggering any positive loops developing their effect in the months that followed.

In addition, there was some variation in the effects of user fee subsidisation on health-care utilisation at one month and 24 months between health zones. Although the majority demonstrated a positive effect at one month, two zones showed a negative effect. This is similar to the findings reported from a study on the abolition of user fees in Zambia and Niger [7], with the authors speculating that negative effects could be explained by varying degrees of enforcement of the new user fee policy; in some areas, informal fees may have continued to be charged at health facilities despite introduction of the policy.

The timing of salary supplements and free drug provision did not appear to modify the effect of user fee subsidisation. This may be explained by the fact that these health system strengthening measures alone are not enough to sufficiently improve health-care utilisation; the addition of user fee subsidisation may further increase rates of health-care utilisation. Indeed, all zones were receiving the full package of interventions by the time user fee subsidisation was introduced, strengthening the argument that all three measures should be in place if significant improvements in health-care utilisation are to be achieved.

One strength of our study is that it took into account potential sources of bias, such as autocorrelation, which often arise in the analysis of routine longitudinal data [25], and used the methodology set out in a recent Cochrane systematic review which concluded that more rigorous research on the effects of user fees on health-care utilisation was needed [6]. Secondly, the study covered a large population over a five year period, thus increasing the statistical power of the results. It is possible that data reporting improved over the life of the programme as health workers were trained over the course of the programme in monitoring and evaluation. Yet, the steady data completion rates over time suggest that this was not a major factor. This study also attempted to control for the confounding effect of salary supplements and drug provision, albeit in only seven health zones.

There were a number of study limitations. Firstly, although the population denominators that were used were the best available, they were nonetheless unreliable as they were extrapolated from a census conducted almost 30 years ago. This is a limitation common to all recent analyses of demographic information in the DRC. The numerator data could also have been manipulated by health zone staff; although there were regular spot checks and audits as well as supervision by the NGOs to ensure data recording was accurate, it is still possible that some zones could have over-reported utilisation figures. The extent to which this may have occurred could not be quantified in this study. Secondly, data disaggregated by age and sex were not available for our study, and so the effect of user fees on different sub-groups of the population could not be assessed. Such data should be collected and analysed in any future user fee subsidy programmes in the DRC. Thirdly, there was some anecdotal evidence that people living in health zones which were not included in the programme were seeking care in health zones with user fee subsidisation, sometimes travelling long distances to receive care. This spill-over effect could not be quantified, and may have inflated our results. However, it also provides evidence that user fees act as a barrier to accessing services, if people are compelled to travel considerable distances to avoid them. In addition, factors which may have affected health-care utilisation at different points over the five year period (e.g. episodes of conflict or displacement, construction work, and training funded by the programme) were not measured as part of this study. This was mitigated by the fact that data on utilisation at least a year prior to and two years after the implementation of user fee subsidisation were available for all health zones allowing reliable trend effects to be established. In the South Kivu health zones in particular, episodes of conflict and displacement occurred periodically between 2008 and 2012, which would have been expected to lead to a concomitant decrease in utilisation rates. However, this was not observed in these health zones. On the other hand, registration for the elections in November 2011 which occurred in health facilities may have driven an increase in utilisation rates in general across health zones during this time.

A major gap in current health policy research is the lack of studies on the impact of user fee subsidisation on mortality and other health indicators. Unfortunately, given the retrospective nature of our study, we were unable to assess the impact of user fee subsidisation on such indicators but further studies exploring this are warranted. Finally, without performing a randomised controlled trial, it is not possible to test the assumption that the underlying trend in utilisation prior to the introduction of the user fee policy would have continued in the absence of the intervention.

While recognising that there are many facets and strategies that need to be considered in terms of the DRC achieving universal health coverage, this study illustrates that the current levels of user fees in the DRC may present a barrier to accessing health-care. It was beyond the scope of this study to consider the issue of sustainability with respect to user fee subsidisation. However, any reluctance to subsidise user fees is largely due to the increased health budget required. As such, for user fee subsidisation to be implemented successfully there has to be long-term commitment and investment by international donors and national governments. Investing in a public health system that is underused because of financial barriers is not an optimal allocation of resources. Until financial barriers are addressed, improvements in health system funding to tackle essential elements such as provision of health-care facilities, inadequate drug supplies and poor quality medications, poor training and motivation of health staff, shortages of health-care workers (all common in poor resource settings), will continue to benefit only a limited number of people. This is of utmost importance for donors and policy makers striving towards health impact. Furthermore, the feasibility of alternative financing mechanisms should also be explored and in this vein, DFID’s new Access to Health-care programme aims to explore and evaluate the effect of a system of community health insurance [31].

## Conclusion

In conclusion, this study adds some evidence that subsidising user fees may increase utilisation in the short-term, and it is hoped that donors and government will embrace these findings when deciding on health financing policy, particularly as there is not yet a consensus view. Future work however should focus on feasibility and sustainability issues associated with the removal or reduction of user fees and how to sustain its effects on utilisation in the longer term [32].
